# On the Mode Localization Between Two Unidentical Resonators with Different Bending Modes for Acceleration Sensing

**DOI:** 10.3390/s25185632

**Published:** 2025-09-10

**Authors:** Bo Yang, Ming Lyu, Jian Zhao, Najib Kacem

**Affiliations:** 1 Transportation Institute, Inner Mongolia University, Hohhot 010021, China; 32424144@mail.imu.edu.cn; 2 Inner Mongolia Engineering Research Center for Intelligent Transportation Equipment, Hohhot 010021, China; 3 School of Mechanical Engineering, Dalian University of Technology, Dalian 116024, China; jzhao@dlut.edu.cn; 4 FEMTO-ST Institute, Université Marie et Louis Pasteur, CNRS, F-25000 Besançon, France; najib.kacem@femto-st.fr

**Keywords:** acceleration sensor, mode localization, electrostatic coupling, nonlinear dynamics

## Abstract

In the research, a novel accelerometer concept leveraging the mode-localization phenomenon is put forward. The sensor measures external acceleration through monitoring changes in the relative amplitude ratio among coupled resonators. The sensing part of the presented accelerometer comprises a doubly clamped beam coupled with a cantilever beam. Its design ensures the initial bending mode of the clamped beam approximates the secondary bending mode of the cantilever. Drawing on Euler–Bernoulli beam theory, the governing formulas of the coupled resonators are deduced and analyzed via Galerkin discretization integrated with the multiple-scale method. During working in both linear as well as nonlinear operating regions, this sensor’s dynamic behavior can be tuned by adjusting the drive voltage. The obtained results demonstrate that the nonlinear dynamics increases the accelerometer sensitivity, which can be further enhanced by adjusting the coupling voltage without severe mode overlap. The presented model offers one viable method to enhance the overall performance in multi-mode MEMS accelerometers.

## 1. Introduction

The micro resonant accelerometer is a category of MEMS sensors that transform inertial input into shifts in resonance frequency [1,2]. High-sensitivity resonant acceleration sensors exhibit extensive demand and considerable application potential within the domain of automotive safety, consumer electronics, aerospace, and intelligent equipment, and continue to play an important role in the development of emerging technologies such as unmanned driving and intelligent manufacturing [3,4,5]. Traditional resonant accelerometers primarily employ a single resonator as the sensing element, detecting changes through its frequency shift. Several studies have reported that operating resonators in higher-order modes can lead to significant improvements in sensitivity [6,7,8,9]. Under the constraints of detection principle and processing technology, it is difficult to enhance the sensitivity of traditional resonant acceleration sensors, which is a key technical problem restricting the application of micro-mechanical resonant acceleration sensors [10].

**Figure 1:**
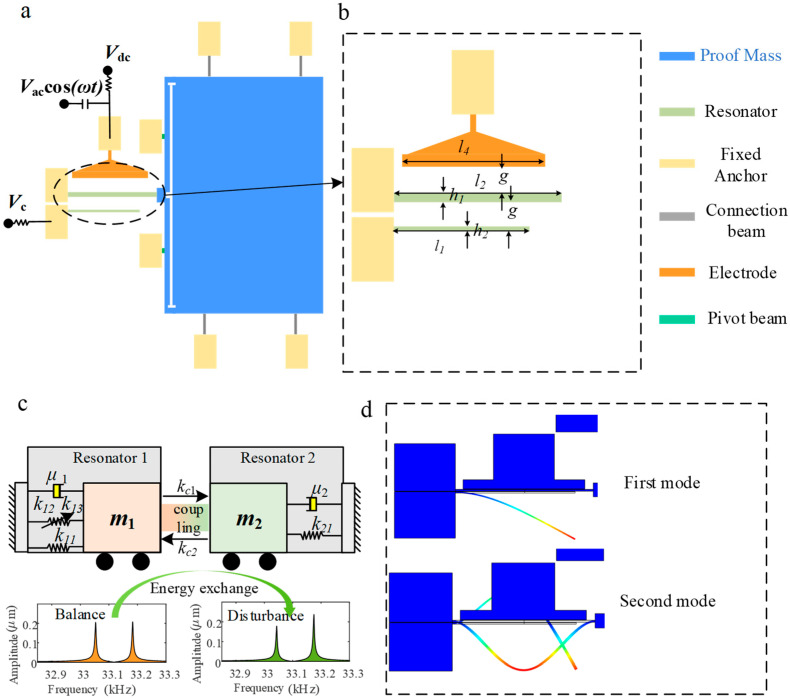
Sketch of the mode-localized accelerometer: (**a**) applied voltage connection line; (**b**) acceleration sensor size structure; (**c**) schematic diagram of a 2-DoF system; (**d**) schematic diagram of the coupled system operating with modes of different orders.

In recent years, the exploration into the theory and application of mode localization has offered a new approach to overcoming the bottlenecks of traditional sensor technology [11]. Since mode localization can significantly improve the sensitivity of resonant sensors by using a new detection mechanism, the schematic diagram of mode localization is shown in Figure 1c. When the system is disturbed, the amplitude undergoes drastic changes. The phenomenon has been rapidly developed in the design and application of acceleration sensors [12,13]. Zhang et al. [14] developed a mode localization-based accelerometer, which utilizes a structure comprising a pair of coupled resonators. Performance was evaluated by comparing the measured relative amplitude proportion against the frequency deviation. Results demonstrate that this occurrence exhibits high sensitivity and robust immunity to disturbances. Zhao et al. [15] proposed a novel modal-localized acceleration sensor incorporating a lever mechanism, which amplifies the axial force applied to the resonator, changing its stiffness and leading to a significant eigenstate variation. It should be noted that the resonators in the described structure are mechanically coupled. While this form of coupling offers excellent stability, it also results in a fixed coupling strength that cannot be adjusted after fabrication, significantly impacting the sensitivity of the device. Thus, to realize tunable coupling intensity and boost the sensitivity in mode-localized sensors, numerous scholars have proposed employing electrostatic coupling [16]. Thiruvenkatanathan et al. [17] first proposed the use of electrostatic coupling in the study of mode localization and they found that the use of such a mechanism can significantly reduce the coupling strength, making it possible for the system to obtain a stronger and tunable mode localization phenomenon. It is noteworthy that electrostatic coupling and stiffness modulation have also been widely investigated in frequency-modulated (FM) resonant accelerometers, where the acceleration-induced stress shift is transduced into a resonant frequency change, demonstrating high resolution and stability [18]. Morozov et al. [19] developed an electrostatically coupled mode-localized accelerometer model, systematically investigated the dependence of resonant frequencies and eigenvector elements with respect to the intensity of inertial effect, and comprehensively analyzed the influence on coupling potential over the characteristic frequency. It was shown that as the coupling potential increases, the frequency disparity at the mode veering point decreases gradually. Peng et al. [20] developed a novel mode-localized acceleration sensor consisting of four resonators. Specifically, resonator 1 and resonator 2 are mechanically linked in series to resonator 3, while resonator 4 is electrostatically coupled to both resonator 1 and resonator 3. Open-loop tests revealed that under varying tuning voltages, the measured amplitude proportion sensitivity ranges, exhibiting a tunable range of up to 2050%. These findings illustrate that the mode localization phenomenon in coupled resonators has achieved substantial maturity in sensor design and applications—with particular relevance to accelerometers.

The aforementioned studies demonstrate that notable outcomes have been attained in the design and practical application of acceleration sensors based on the mode localization phenomenon in coupled resonators. However, it should also be noted that most of these research studies use mainly linearized models for dynamics analysis. In fact, there is a shortage of effective research regarding the influence of nonlinear force terms in coupled resonators. Zhang et al. [21] experimentally investigated the nonlinear behavior of a mode-localized accelerometer. By elevating the excitation voltage to operate the sensor in the nonlinear regime, they employed shifts in the bifurcation point for acceleration detection. In addition, temperature control was adopted to reduce phase and amplitude variations caused by thermal drift, which significantly decreased 1/f noise at the operational frequency. Lyu et al. [22] proposed a modal localization phenomenon and applied it to the design of an accelerometer sensor integrating a lever and a proof mass and investigated its sensitivity change under nonlinear vibration.

However, when it comes to applying the mode localization effect within sensors, the majority of the proposed structures are made up of coupled identical or near-identical resonators, we investigate in this paper a new configuration that couples two different resonators and exploits the coupling between different order modes to further expand the application of this phenomenon in sensors. To address this gap, a new accelerometer configuration utilizing the mode localization effect is proposed, comprising a sensing element consisting of a clamped-clamped beam linked to a cantilever beam. The proposed sensor exploits the phenomenon of modal coupling that arises between modes of different orders in a coupled system and the magnitude of the acceleration can be obtained by detecting the relative amplitude ratio between the two different resonators. First, drawing on Euler–Bernoulli beam theory, the motion equations of the coupled system are derived (incorporating both electrostatic and geometric nonlinearities) and solved via Galerkin discretization combined with the method of multiple time scales; next, the coupled system is switched into linear and nonlinear states by adjusting the AC voltage, and its sensitivity under each state is explored; finally, the influence in coupling voltage variations upon the dynamic behavior and performance of the designed sensor is investigated. It is important to note that this study primarily serves as a theoretical and numerical investigation into a novel accelerometer concept. The primary aim is to establish a foundational model and explore the potential performance benefits, particularly the significant enhancement in sensitivity, offered by coupling dissimilar resonators and exploiting nonlinear dynamics.

## 2. Design and Model

### 2.1. Accelerometer Design and Working Principle

Figure 1a presents a schematic diagram of the proposed accelerometer design. Unlike traditional mode-localized accelerometers, the sensing component consists of two electrostatically coupled resonators with distinct characteristics, as shown in Figure 1d. Specifically, this design explicitly refers to the coupling between the primary-order mode in a doubly clamped beam and the secondary-order mode in a cantilever beam. The cantilever beam is electrostatically coupled to the clamped-clamped beam. The latter is actuated via an electrode on which DC and AC voltages are superimposed. The proof mass is mechanically supported on its left side by a clamped beam, which connects it to a stationary anchor. When external acceleration along the horizontal axis is applied, the proof mass undergoes axial displacement. This displacement changes the axial stress in the clamped beam, which in turn disrupts the equilibrium of the fully coupled system. The proposed design allows acceleration detection by measuring the amplitude shift between the two coupled resonators.

### 2.2. Dynamic Model

Drawing on Euler–Bernoulli theory and under the assumption that the geometric nonlinearity of the cantilever is negligible compared to the mechanical nonlinearity in the clamped-clamped beam, the governing motion equations for the coupled-resonator system are formulated as below [23]:

(1) $$\begin{array}{l} \frac{Ebh_{1}^{3}}{12}\frac{\partial^{4}{\tilde{w}}_{1}\left(\tilde{x},\tilde{t}\right)}{\partial {\tilde{x}}^{4}}+\rho bh_{1}\frac{\partial^{2}{\tilde{w}}_{1}\left(\tilde{x},\tilde{t}\right)}{\partial {\tilde{t}}^{2}}+{\tilde{c}}_{1}\frac{\partial {\tilde{w}}_{1}\left(\tilde{x},\tilde{t}\right)}{\partial \tilde{t}}-\left[\tilde{N}+\frac{Ebh_{1}}{2l_{1}}\int_{0}^{l_{1}}\frac{\partial {\tilde{w}}_{1}{\left(\tilde{x},\tilde{t}\right)}^{2}}{\partial \tilde{x}}d\tilde{x}\right]\frac{\partial^{2}{\tilde{w}}_{1}\left(\tilde{x},\tilde{t}\right)}{\partial {\tilde{x}}^{2}}\\ +\frac{\varepsilon_{0}b{V_{c}}^{2}H_{2}\left(\tilde{x}\right)}{2{\left(g+{\tilde{w}}_{1}\left(\tilde{x},\tilde{t}\right)-{\tilde{w}}_{2}\left(\tilde{x},\tilde{t}\right)\right)}^{2}}=\frac{1}{2}\varepsilon_{0}bH_{1}\left(\tilde{x}\right)\frac{{\left(V_{dc}+V_{ac}\cos\left(\tilde{\Omega }\tilde{t}\right)\right)}^{2}}{{\left(g-{\tilde{w}}_{1}\right)}^{2}}\\ \frac{Ebh_{2}^{3}}{12}\frac{\partial^{4}{\tilde{w}}_{2}\left(\tilde{x},\tilde{t}\right)}{\partial {\tilde{x}}^{4}}+\rho bh_{2}\frac{\partial^{2}{\tilde{w}}_{2}\left(\tilde{x},\tilde{t}\right)}{\partial {\tilde{t}}^{2}}+{\tilde{c}}_{2}\frac{\partial {\tilde{w}}_{2}\left(\tilde{x},\tilde{t}\right)}{\partial \tilde{t}}-\frac{\varepsilon_{0}b{V_{c}}^{2}H_{2}\left(\tilde{x}\right)}{2{\left(g+{\tilde{w}}_{1}\left(\tilde{x},\tilde{t}\right)-{\tilde{w}}_{2}\left(\tilde{x},\tilde{t}\right)\right)}^{2}}=0 \end{array}$$

where *H*_1_ and *H*_2_ are Heaviside functions

(2) $$\begin{array}{l} H_{1}\left(\tilde{x}\right)=\left|H\left(\tilde{x}-\frac{l_{1}-l_{4}}{2}\right)-H\left(\tilde{x}-\frac{l_{1}+l_{4}}{2}\right)\right|\\ H_{2}\left(\tilde{x}\right)=H\left(\frac{l_{2}}{2}-\tilde{x}\right) \end{array}$$

Moreover, the boundary conditions for the microbeams are prescribed as follows:

(3) $$\left\{\begin{array}{l} {\tilde{w}}_{1}\left(0,\tilde{t}\right)={\tilde{w}}_{1}\left(l_{1},\tilde{t}\right)=\frac{\partial {\tilde{w}}_{1}}{\partial \tilde{x}}\left(0,t\right)=\frac{\partial {\tilde{w}}_{1}}{\partial \tilde{x}}\left(l_{1},\tilde{t}\right)=0\\ {\tilde{w}}_{2}\left(0,\tilde{t}\right)=\frac{\partial {\tilde{w}}_{2}}{\partial \tilde{x}}\left(0,\tilde{t}\right)=\frac{\partial^{2}{\tilde{w}}_{2}}{\partial {\tilde{x}}^{2}}\left(l_{2},t\right)=\frac{\partial^{3}{\tilde{w}}_{2}}{\partial {\tilde{x}}^{3}}\left(l_{2},\tilde{t}\right)=0 \end{array}\right.$$

where ${\tilde{w}}_{1},{\tilde{w}}_{2}$ represent the transverse deflection of the two resonators along the *x* axis, ${\tilde{N}}_{1}$ denotes the mechanical axial force, *l*_1_, *l*_2_, *h*_1_, *h*_2_, and b stand for the length, thickness, and width corresponding to the two resonators, *ρ* represents the material density, *ε*_0_ the dielectric constant, *E* stands for Young’s modulus, *g* the capacitor gaps, *V_ac_* the alternating current (AC) drive voltage, *V_dc_* the direct current (DC) biasing voltage, Ω the excitation frequency.

## 3. Solving Procedure

### 3.1. Nondimensionalization

For the sake of convenience, Equation (1) can be normalized by means of the following dimensionless parameters.

(4) $$w_{1}=\frac{{\tilde{w}}_{1}}{g},w_{2}=\frac{{\tilde{w}}_{2}}{g},x=\frac{\tilde{x}}{l_{1}},t=\tilde{t}\sqrt{\frac{Eh_{1}^{2}}{12\rho {l_{1}}^{4}}}$$

substituting the dimensionless variable (4) into (1), the following coupled equations are derived:

(5) $$\left\{\begin{array}{l} \frac{\partial^{4}w_{1}}{\partial x^{4}}+\frac{\partial^{2}w_{1}}{\partial t^{2}}+c_{1}\frac{\partial w_{1}}{\partial t}-\left[N+\alpha_{1}\int_{0}^{1}{\left(\frac{\partial w_{1}}{\partial x}\right)}^{2}dx\right]\frac{\partial^{2}w_{1}}{\partial x^{2}}\\ =\frac{\alpha_{2}H_{1}\left(x\right){\left(V_{\mathrm{dc}}+V_{\mathrm{ac}}\cos(\Omega t)\right)}^{2}}{{\left(1-w_{1}\right)}^{2}}-\frac{\alpha_{2}H_{2}\left(x\right){V_{c}}^{2}}{{\left(1+w_{1}-w_{2}\right)}^{2}}\\ \delta^{2}\frac{\partial^{4}w_{2}}{\partial x^{4}}+\frac{\partial^{2}w_{2}}{\partial t^{2}}+c_{2}\frac{\partial w_{2}}{\partial t}=\frac{\alpha_{2}H_{2}\left(x\right){V_{c}}^{2}}{{\left(1+w_{1}-w_{2}\right)}^{2}} \end{array}\right.$$

The dimensionless coefficients in Equation (5) are given by

(6) $$\begin{array}{l} c_{1}=\frac{12\tilde{c}l_{1}^{4}}{Ebh_{1}^{3}\tau_{1}},c_{2}=\frac{12\tilde{c}l_{1}^{4}}{Ebh_{2}h_{1}^{2}\tau_{1}},N=\frac{12\tilde{N}l_{1}^{2}}{Ebh_{1}^{3}},\alpha_{1}=6{\left(\frac{g}{h_{1}}\right)}^{2}\\ \alpha_{2}=\frac{6\varepsilon_{0}l_{1}^{4}}{Eh_{1}^{3}g^{3}},\delta =\frac{h_{2}}{h_{1}},\tau_{1}=\sqrt{\frac{12\rho l_{1}^{4}}{Eh_{1}^{2}}},\Omega =\tilde{\Omega }\tau \end{array}$$

### 3.2. Nonlinear Reduced Order Model

Through Galerkin discretization, the partial differential equation system in Equation (5) is reduced to a set of ordinary differential equations. Given the weak inter-resonator coupling, the linear mode function of an undamped straight beam is adopted as the basis for discretization. An approximate solution to the dimensionless dynamic equation is formulated as follows:

(7) $$\left\{\begin{array}{l} w_{1}\left(x,t\right)=w_{s1}\left(x\right)+\sum_{j=1}^{N_{m}}q_{1,j}\left(t\right)\phi_{1,j}\left(x\right)\\ w_{2}\left(x,t\right)=w_{s2}\left(x\right)+\sum_{j=1}^{N_{m}}q_{2,j}\left(t\right)\phi_{2,j}\left(x\right) \end{array}\right.$$

where $w_{s1}\left(x\right)$, $w_{s2}\left(x\right)$ denote the static displacement of the clamed-clamped beam and the cantilever, respectively, and *ϕ*_1,*j*_(*x*), *ϕ*_2,*j*_(*x*) represent the *j*-order mode of the corresponding resonator, respectively, based on the bending vibration of the beam, according to the boundary conditions.

While assuming that only a single bending mode is dominant for each resonator and the other modes are neglected and neglecting the effects of higher modes (*N_m_* = 1) [24], the dynamic formulas for the system may be derived as below:

(8) $$\left\{\begin{array}{l} {\ddot{q}}_{1}+\mu_{1}{\dot{q}}_{1}+\kappa_{11}^{2}q_{1}+\kappa_{12}q_{1}^{2}+\kappa_{13}q_{1}^{3}+\kappa_{c1}q_{1}+\kappa_{c2}q_{2}+f_{1}\cos\left(\Omega t\right)=0\\ {\ddot{q}}_{2}+\mu_{2}{\dot{q}}_{2}+\kappa_{21}^{2}q_{2}-\kappa_{c1}q_{1}-\kappa_{c2}q_{2}=0 \end{array}\right.$$

where a dot denotes the time derivative. The resulting differential system corresponds to a forced Duffing equation—incorporating quadratic and cubic nonlinearities—that is coupled to a linear oscillator. The parameters of the generated reduced order model are given in Appendix A.

### 3.3. Static Analysis

When a micro-resonator is electrostatically driven, exceeding a critical driving voltage can cause the resonator to pull in toward the fixed electrode, leading to a short circuit and eventual device failure. To prevent this static pull-in phenomenon, it is necessary to analyze the static deformation of the electrostatically driven resonator and determine the pull-in voltage threshold under the specified parameters.

**Figure 2:**
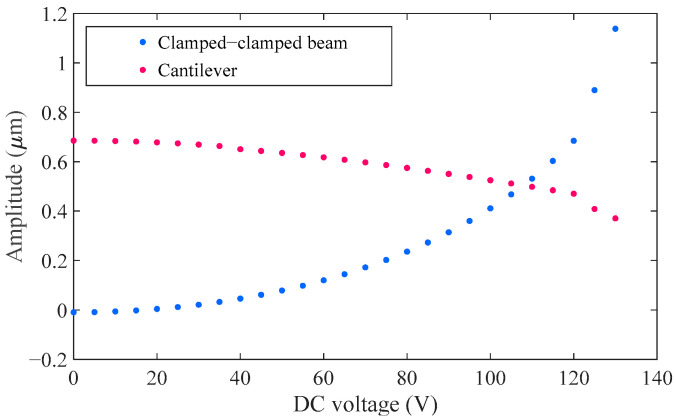
Static analysis simulation results.

The relationship between the static displacement of the two resonators and the applied voltage is illustrated in Figure 2. For the clamped–clamped beam, the maximum deformation occurs at the midpoint, whereas for the cantilever beam, it occurs at the free end. As the DC bias voltage (Vdc) increases, the deformation of the clamped–clamped beam grows progressively, bringing it closer to the fixed drive electrode. In contrast, the cantilever beam is influenced only by electrostatic coupling forces and therefore does not experience collapse. At a drive voltage of approximately 130 V, the clamped–clamped beam adheres to the electrode, indicating pull-in. Accordingly, the safe operating voltage should remain below 130 V.

### 3.4. Eigenvalue Analysis

**Figure 3:**
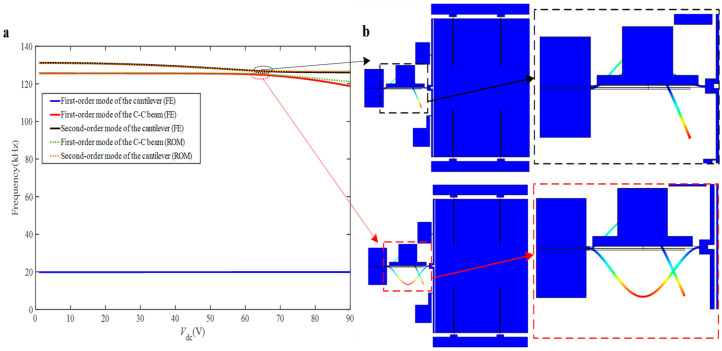
(**a**) The natural frequency variation with the V*_dc_*; (**b**) model established in COMSOL Multiphysics.

Mode veering is an important phenomenon characterizing mode localization. Thus, the existence of mode turning is explored in this coupled system by performing an eigenvalue analysis. As illustrated in Figure 3a, the eigenfrequencies of the coupled system change with respect to the driving voltage V*_dc_*, and remarkably the second-order mode of the cantilever beam and the first-order mode of the doubly clamped undergo mode veering as V*_dc_* keep increasing, which proves that the structure can produce mode localization phenomenon. Figure 3b shows the two vibration modes of the structure at the veering point, obtained using the Reduced Order Model (ROM) and verified by Finite Element (FE) simulations implemented in COMSOL Multiphysics 5.6. The out-of-phase and in-phase modes exhibit resonant frequencies of 125.27 kHz and 126.86 kHz, respectively. When the direct current V*_dc_* reaches 64 V, the two eigenfrequencies reach the veering point, and then, the two modes gradually move away from each other with the increase of V*_dc_*. Consequently, the proposed coupled structure can be used as a sensitive part based on mode localization for acceleration sensing.

### 3.5. Nonlinear Frequency Response

The nonlinear dynamic behavior in the designed structural design is analyzed via the method of multiple scales.

(9) $$\left\{\begin{array}{l} {\ddot{q}}_{1}+\varepsilon^{2}\mu_{1}{\dot{q}}_{1}+\kappa_{11}^{2}q_{1}+\varepsilon \kappa_{12}q_{1}^{2}+\varepsilon^{2}\kappa_{13}q_{1}^{3}+\varepsilon^{2}\kappa_{c1}q_{1}+\varepsilon^{2}\kappa_{c2}q_{2}+\varepsilon^{2}f_{1}\cos\left(\Omega t\right)=0\\ {\ddot{q}}_{2}+\varepsilon^{2}\mu_{2}{\dot{q}}_{2}+\kappa_{21}^{2}q_{2}-\varepsilon^{2}\kappa_{c1}q_{1}-\varepsilon^{2}\kappa_{c2}q_{2}=0 \end{array}\right.$$

the relationship between the different scale times *T_n_* is

(10) $$T_{n}=\varepsilon^{n}t\left(n=0,1,2\right)$$

(11) $$\begin{array}{l} \frac{d}{dt}=\frac{dT_{0}}{dt}\frac{\partial }{\partial T_{0}}+\frac{dT_{1}}{dt}\frac{\partial }{\partial T_{1}}+\frac{dT_{2}}{dt}\frac{\partial }{\partial T_{2}}+\ldots =D_{0}+\varepsilon D_{1}+\varepsilon^{2}D_{2}+\ldots \\ \frac{d^{2}}{{\mathrm{dt}}^{2}}=D_{0}^{2}+2\varepsilon D_{0}D_{1}+\varepsilon^{2}\left(D_{1}^{2}+2D_{0}D_{2}\right)+\ldots \end{array}$$

the detuning parameters *σ*_1_ and *σ*_2_ are introduced to characterize the motion near the resonant frequencies.

(12) $$\begin{array}{l} \Omega =\kappa_{11}+\varepsilon^{2}\sigma_{1}\\ \kappa_{11}=\kappa_{21}+\varepsilon^{2}\sigma_{2} \end{array}$$

the solutions of Equation (9) are assumed as follows

(13) $$\begin{array}{l} q_{1}=q_{10}\left(T_{0},T_{1},T_{2}\right)+\varepsilon q_{11}\left(T_{0},T_{1},T_{2}\right)+\varepsilon^{2}q_{12}\left(T_{0},T_{1},T_{2}\right)\\ q_{2}=q_{20}\left(T_{0},T_{1},T_{2}\right)+\varepsilon q_{21}\left(T_{0},T_{1},T_{2}\right)+\varepsilon^{2}q_{22}\left(T_{0},T_{1},T_{2}\right) \end{array}$$

substituting Equation (13) into Equation (9), the following system of equations is generated based on the order of *ε*

(14) $$\begin{array}{ll} \varepsilon^{0}: & D_{0}^{2}q_{10}+\kappa_{11}^{2}q_{10}=0\\ & D_{0}^{2}q_{20}+\kappa_{21}^{2}q_{20}=0 \end{array}$$

(15) $$\begin{array}{ll} \varepsilon^{1}: & \kappa_{11}^{2}q_{11}+D_{0}\left(D_{0}q_{11}+D_{1}q_{10}\right)+D_{1}D_{0}q_{10}=-\kappa_{12}q_{10}^{2}\\ & \kappa_{21}^{2}q_{21}+D_{0}\left(D_{0}q_{21}+D_{1}q_{20}\right)+D_{1}D_{0}q_{20}=0 \end{array}$$

(16) $$\begin{array}{ll} \varepsilon^{2}: & D_{0}\left(D_{0}q_{12}+D_{1}q_{11}+D_{2}q_{10}\right)+D_{1}\left(D_{0}q_{11}+D_{1}q_{10}\right)\\ & +D_{2}D_{0}q_{10}+\kappa_{11}^{2}q_{12}=-cD_{0}q_{10}-\left(-\kappa_{c1}q_{10}+\kappa_{c2}q_{20}\right)\\ & -\kappa_{13}q_{10}^{3}-\kappa_{12}q_{10}q_{11}-f_{1}\cos\left(\kappa_{11}T_{0}+\sigma_{1}T_{2}\right)\\ & D_{0}\left(D_{0}q_{22}+D_{1}q_{21}+D_{2}q_{20}\right)+D_{1}\left(D_{0}q_{21}+D_{1}q_{20}\right)\\ & +D_{2}D_{0}q_{20}+\kappa_{21}^{2}q_{22}=-cD_{0}q_{20}+\left(\kappa_{c1}q_{10}+\kappa_{c2}q_{20}\right) \end{array}$$

The solutions of Equation (14) can be defined as

(17) $$\begin{array}{l} q_{10}=X_{1}\exp\left(i\kappa_{11}T_{0}\right)+\bar{X_{1}}\exp\left(-i\kappa_{11}T_{0}\right)\\ q_{20}=X_{2}\exp\left(i\kappa_{21}T_{0}\right)+\bar{X_{2}}\exp\left(-i\kappa_{21}T_{0}\right) \end{array}$$

where *X*_1_ and *X*_2_ are complex functions (with their complex conjugates denoted as) and employing the subsequent polar form for representation:

(18) $$X_{1}=\frac{1}{2}A_{1}e^{i\beta_{1}},X_{2}=\frac{1}{2}A_{2}e^{i\beta_{2}}$$

where *A*_1_ and *A*_2_ denote the amplitudes of resonators 1 and 2, respectively. By substituting Equation (18) into Equations (15) and (16), and separating the real and imaginary components, we derive this result.

(19) $$\left\{\begin{array}{l} \sin\left(\varphi_{1}\right)=\frac{-A_{1}\kappa_{11}\mu_{1}+\frac{{A_{2}}^{2}\kappa_{21}\kappa_{c2}\mu_{2}}{A_{1}\kappa_{c1}}}{f_{1}}\\ \cos\left(\varphi_{1}\right)=\frac{10{A_{1}}^{3}\kappa_{12}^{2}-9{A_{1}}^{3}\kappa_{11}^{2}\kappa_{13}-12A_{1}\kappa_{11}^{2}\kappa_{c1}+24A_{1}\kappa_{11}^{3}\sigma_{1}-\cos\left(\varphi_{2}\right)}{12f_{1}\kappa_{11}^{2}}\\ \sin\left(\varphi_{2}\right)=-\frac{A_{2}\kappa_{21}\mu_{2}}{A_{1}\kappa_{c1}}\\ \cos\left(\varphi_{2}\right)=-\frac{A_{2}\left(\kappa_{c2}+2\kappa_{21}\sigma_{1}+2\kappa_{21}\sigma_{2}\right)}{A_{1}\kappa_{c1}} \end{array}\right.$$

## 4. Numerical Results and Discussion

Unlike traditional resonant sensors, which rely on frequency shift as the output, the mode-localization sensor employs the variation in the amplitude ratio as its readout metric. To enable a unified comparison of these two output metrics, the relative shifts in frequency and amplitude ratio are defined as follows:

(20) $$S_{f}=\left(\omega_{1,1}-\omega_{1,1}^{0}\right)/\omega_{1,1}^{0}$$

(21) $$S_{a}=\left(\frac{w_{1,2}}{w_{2,2}}-\frac{w_{1,2}^{0}}{w_{2,2}^{0}}\right)/\frac{w_{1,2}^{0}}{w_{2,2}^{0}}$$

where *S_f_* and *S_a_* denote the sensitivities defined as the ratios of the relative frequency shift and the relative amplitude ratio, *ω_i_*,*_j_*^0^ represents the natural frequency of the *j*th order mode for the *i*th resonator in the balanced state, *ω_i_*,*_j_* is the frequency after stiffness perturbation on the clamped-clamped resonator. *w*_1,1_ and *w*_1,2_ are the amplitudes of the resonators post stiffness disturbance, while *w*_1,1_^0^ and *w*_2,2_^0^ refer to the amplitudes of the two resonators in the balanced condition.

### 4.1. Linear Behavior

**Figure 4:**
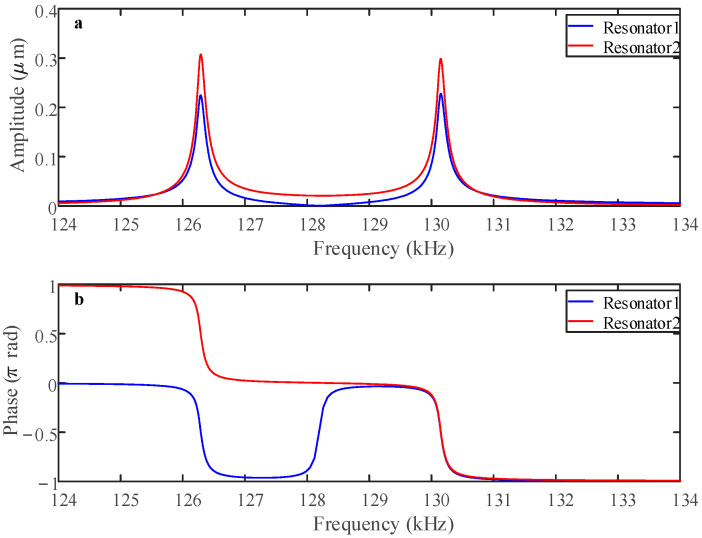
Linear (**a**) amplitude and (**b**) phase frequency responses for V*_c_* = 70 V, V*_dc_* = 62 V, and V*_ac_* = 0.1 V when the sensor is not subject to an acceleration disturbance.

Drawing on the parameters listed in Table 1, this study examines the amplitude response under balanced conditions. When the AC voltage V*_ac_* = 0.1 V, the coupling voltage V*_c_* = 70 V, and the driving voltage V*_dc_* = 62 V, as shown in Figure 4a, both resonators operate in a linear regime, and the phase variations in the two resonators are shown in Figure 4b. The two electrostatically coupled resonators exhibit out-of-phase vibration in the first mode, whereas they demonstrate in-phase motion in the second mode.

**Figure 5:**
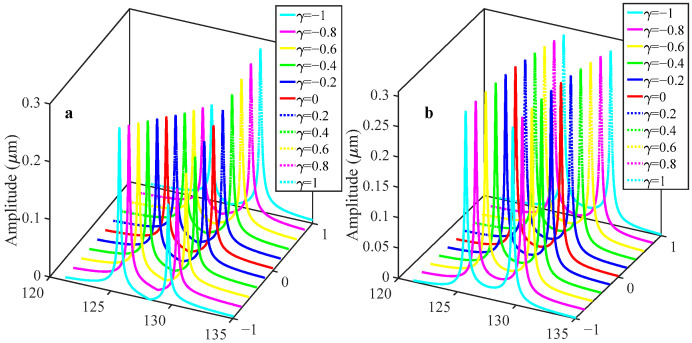
Variation in the amplitude response with respect to the acceleration disturbance when the sensor is driven in the linear regime for V*_c_* = 70 V, V*_dc_* = 62 V, and V*_ac_* = 0.1 V: (**a**) resonator 1; (**b**) resonator 2.

As illustrated in Figure 5, when the applied acceleration varies between −1 g and 1 g, the amplitude value of Resonator 2 increases monotonically as acceleration changes in both operating modes, whereas Resonator 1 exhibits a distinct trend. Specifically, in the in-phase mode, Resonator 1’s amplitude decreases as acceleration rises; conversely, in the out-of-phase operating mode, its amplitude rises correspondingly.

**Figure 6:**
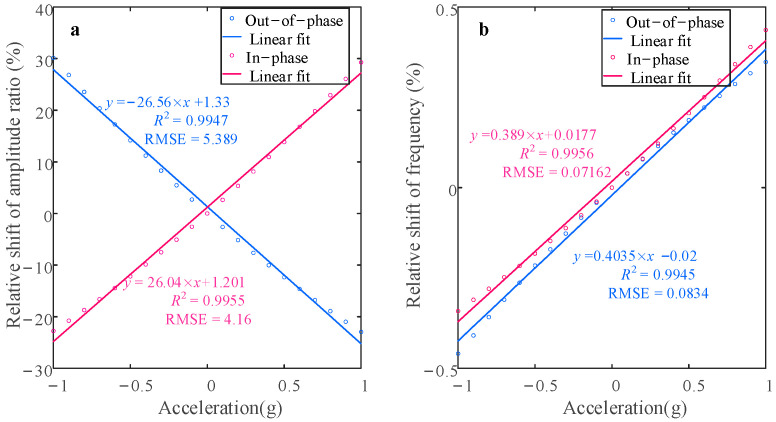
Sensitivity change in different modes when the sensor is driven in the linear regime for V*_c_* = 70 V, V*_dc_* = 62 V, and V*_ac_* = 0.1 V: (**a**) relative shift in amplitude ratio; (**b**) relative shift in frequency.

Figure 6 depicts the variation in the two sensitivity metrics—defined by Equations (20) and (21)—over the acceleration range −1 g to +1 g. The relative frequency shift (RSF) is 0.43%/g in the in-phase mode, decreasing to 0.35%/g in the out-of-phase mode. In contrast, the relative amplitude ratio shift (RSAR) reaches 29.23%/g (in-phase) and 30.13%/g (out-of-phase). Compared to RSF, RSAR exhibits a two-order-of-magnitude sensitivity enhancement.

### 4.2. Nonlinear Behavior

**Figure 7:**
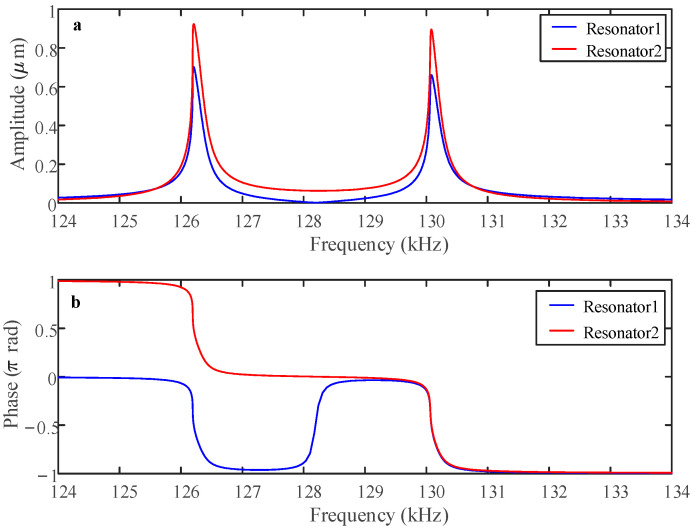
Nonlinear (**a**) amplitude and (**b**) phase frequency responses for V*_c_* = 70 V, V*_dc_* = 62 V, and V*_ac_* = 0.3 V when the sensor is not subject to an acceleration disturbance.

At low AC driving voltages, the vibration amplitude of both resonators remains limited, resulting in a linear frequency response. However, as the AC drive voltage increases, the vibration amplitude gradually rises until it enters the nonlinear domain. When the coupling voltage V*_c_* = 70 V is constant and the AC voltage is raised as high as V*_ac_* = 0.3 V, as shown in Figure 7a, both resonators operate in a nonlinear region and demonstrate spring-softening behavior. The vibrational amplitude demonstrates a marked increase relative to the linear operational regime.

**Figure 8:**
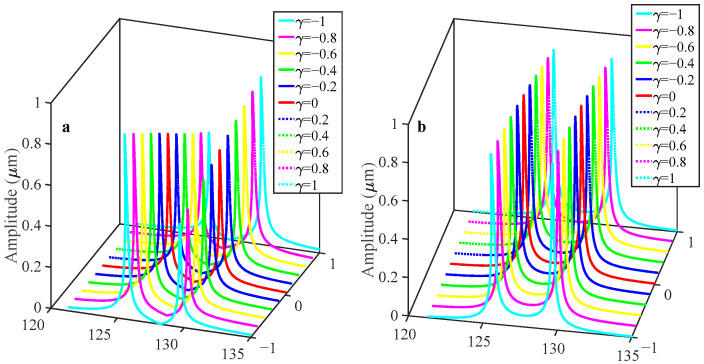
Variation in the amplitude response with respect to the acceleration disturbance when the sensor is driven in the nonlinear regime for V*_c_* = 70 V, V*_dc_* = 62 V, and V*_ac_* = 0.3 V: (**a**) resonator 1; (**b**) resonator 2.

**Figure 9:**
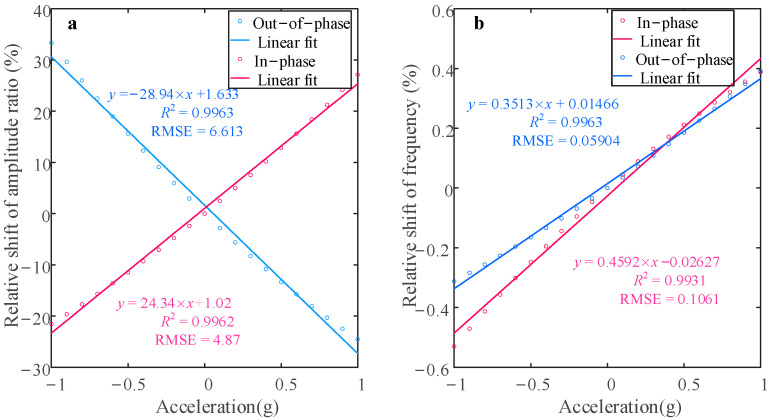
Sensitivity change in different modes when the sensor is driven in the nonlinear regime for V*_c_* = 70 V, V*_dc_* = 62 V, and V*_ac_* = 0.3 V: (**a**) relative shift in amplitude ratio; (**b**) relative shift in frequency.

As shown in Figure 8, when the perturbation acceleration is added in the range [−1 g, 1 g], the form of change in this system in the nonlinear state is consistent with the trend of change in the linear state. When using the same acceleration perturbation range as when the system is in a linear state, the two sensitivity outputs are shown in Figure 9. It can be found that in the in-phase mode, the RSF is 0.38%/g, while the RSAR is 27.11%/g, which rises to 33.34%/g in the out-of-phase mode. The sensitivity, as defined by the relative amplitude ratio in the out-of-phase mode, exhibits a notable enhancement when compared to the linear vibration regime. As shown in Figure 9, when the resonators are subject to nonlinear behavior, the residuals for the in-phase and out-of-phase modes, as determined by linear fitting, are 4.87 and 6.613, respectively. Compared to linear behavior, an increase in residuals can be observed, which can be explained by an increase in the nonlinearity on the scale factor.

Table 2 compares the sensitivity of the proposed accelerometer (in both linear and nonlinear regimes) with other state-of-the-art mode-localized accelerometers reported in the recent literature. Although a direct experimental comparison is not yet available, the simulated sensitivity of our device demonstrates competitive potential, particularly noting that the achieved sensitivity is obtained with a simple two-resonator structure and tunable via the coupling voltage.

### 4.3. Effect of the Coupling Voltage on Sensitivity

**Figure 10:**
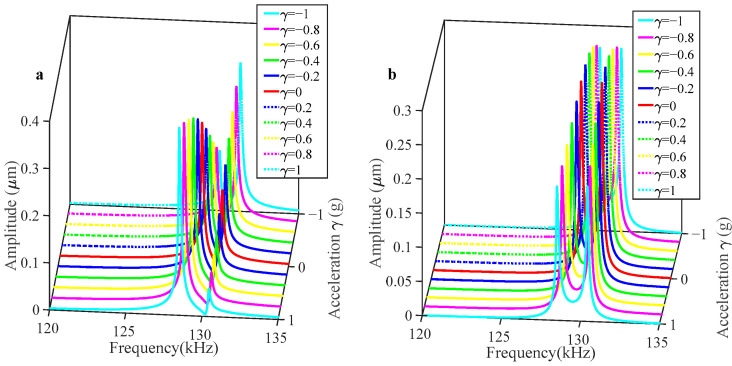
Variation in the amplitude response with respect to the acceleration disturbance for V*_c_* = 40 V, V*_dc_* = 62 V, and V*_ac_* = 0.1 V: (**a**) resonator 1; (**b**) resonator 2.

**Figure 11:**
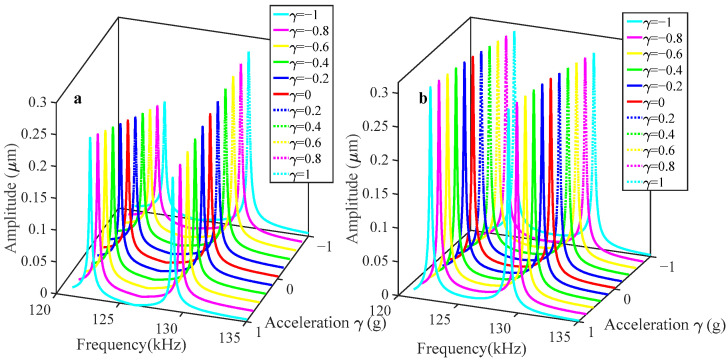
Variation in the amplitude response with respect to the acceleration disturbance for V*_c_* = 90 V, V*_dc_* = 62 V, and V*_ac_* = 0.1 V: (**a**) resonator 1; (**b**) resonator 2.

**Figure 12:**
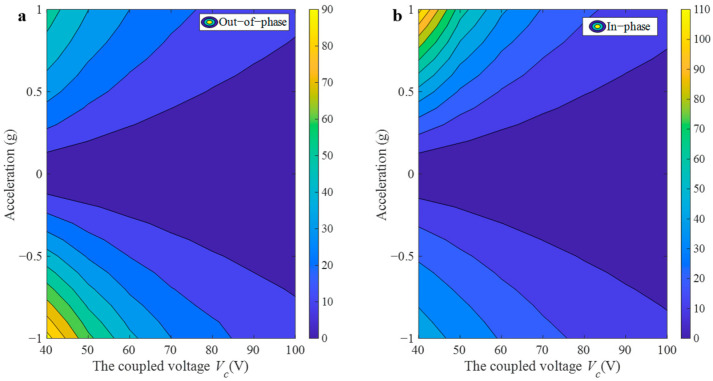
Variation in the relative shift in amplitude ratio with respect to the acceleration disturbance and the coupling voltage for V*_dc_* = 62 V, and V*_ac_* = 0.1 V: (**a**) out-of-phase; (**b**) in-phase.

The variation in coupling voltage V*_c_* is studied and the accelerometer sensitivity is numerically analyzed. As the coupling voltage rises, the coupling strength gradually increases. To further explore the effect of the coupling voltage on the sensitivity in the mode-localization accelerometer, V*_c_* is gradually increased while setting V*_ac_* = 0.1 V and V*_dc_* = 62 V. The sensitivity changes are compared and analyzed, as shown in Figure 10, Figure 11 and Figure 12. The coupling voltage V*_c_* influences the natural frequency of both resonant modes. Since as the coupling voltage increases, the driving force grows, and accordingly, the intrinsic frequency decreases—owing to its dependence on electrostatic negative stiffness.

Figure 12 illustrates that the relative amplitude ratio shift decreases with increasing V*_c_*. For instance, at a coupling voltage V*_c_* of 40 V, the RSAR in out-of-phase mode is 94%, at a voltage of 100 V, this value drops to 13.65%. The sensitivity similarly decreases with increasing coupling strength when the sensor operates in the in-phase mode. Comparing Figure 10 and Figure 11, it can be found that although the reduction in the coupling voltage serves to enhance the sensitivity, the two modes will be gradually close to each other with the reduction of V*_c_*, which will result in a modal coupling without severe mode overlap.

## 5. Conclusions

In this work, the proposed accelerometer operates via mode localization, featuring a doubly clamped beam coupled with a cantilever beam. Focusing on the coupling between the initial mode of the doubly clamped beam and the secondary mode of the cantilever beam, the Euler–Bernoulli equation of motion for the system was derived. The sensor’s linear and nonlinear responses are controlled by an AC voltage on the drive electrode. Simulations revealed a more pronounced out-of-phase mode compared to linear vibration. By measuring the relative amplitude ratio between the two beams, the applied acceleration is determined. This approach achieves a 67-fold sensitivity enhancement over frequency-shift-based detection. Additionally, the coupling voltage’s influence on sensitivity was investigated, revealing that sensitivity is tunable via coupling voltage variation. Specifically, reducing the coupling voltage enhances sensitivity: numerical simulations show an 80.35% increase when the drive voltage decreases from 100 V down to 40 V. In order to translate this theoretical concept into a real device, future work will focus on experimental validation through SOI-MEMS manufacturing, characterization under vacuum, and calibration with precision instrumentation.

## Figures and Tables

**Table 1:** Design parameters of the proposed accelerometer.

Type	Value
Length of resonator (*l_1_*)	905 μm
Length of resonator (*l*_2_)	791 μm
Resonator width (*h*_1_)	12 μm
Resonator width (*h*_2_)	9 μm
Young’s modulus (*E*)	169 GPa
Air gap (*g*)	3 μm
Resonator width (*b*)	25 μm
Density (*ρ*)	2320 kg/m^3^

**Table 2:** Comparing sensitivity performance of different sensor designs.

Reference (Device Type)	Sensitivity (RSAR)	Detection Mechanism
Zhang et al. [14] (4 resonators)	1.14–23.37%/g	Linear Mode Localization
Peng et al. [20] (Series-Parallel)	~20%/g (tunable)	Linear Mode Localization
This work (Linear, Out-of-phase)	30.13%/g	Linear Mode Localization
This work (Nonlinear, Out-of-phase)	33.34%/g	Nonlinear Mode Localization
Zhang et al. [21] (Nonlinear)	~0.1%/g (Bifurcation)	Nonlinear Bifurcation

## Data Availability

Data are contained within the article.

## Appendix A

(A1)
$$\begin{array}{ll} \kappa_{11}= & \lambda_{1,1}^{4}-N\overset{1}{\underset{0}{\int }}\phi_{1,1}^{\prime \prime }\phi_{1,1}dx-2\alpha_{2}V_{\mathrm{dc},1}^{2}\overset{1}{\underset{0}{\int }}H_{1}\phi_{1,1}^{2}dx-6\alpha_{2}V_{\mathrm{dc},1}^{2}\overset{1}{\underset{0}{\int }}H_{1}\phi_{1,1}^{2}w_{s,1}dx\\ & -12\alpha_{2}V_{\mathrm{dc},1}^{2}\overset{1}{\underset{0}{\int }}H_{1}\phi_{1,1}^{2}{w_{s,1}}^{2}dx+\alpha_{1}\overset{1}{\underset{0}{\int }}{w_{s,1}^{\prime }}^{2}dx\overset{1}{\underset{0}{\int }}\phi_{1,1}^{\prime \prime }\phi_{1,1}dx+2\alpha_{1}\overset{1}{\underset{0}{\int }}w_{s,1}^{\prime \prime }\phi_{1,1}dx\overset{1}{\underset{0}{\int }}\phi_{1,1}^{\prime }w_{s,1}^{\prime }dx\\ \kappa_{12}= & -3\alpha_{2}V_{\mathrm{dc},1}^{2}\overset{1}{\underset{0}{\int }}H_{1}\phi_{1,1}^{3}dx+\alpha_{1}\overset{1}{\underset{0}{\int }}w_{s,1}^{\prime \prime }\phi_{1,1}dx\overset{1}{\underset{0}{\int }}{\left(\phi_{1,1}^{\prime }\right)}^{2}dx\\ & +2\alpha_{1}\overset{1}{\underset{0}{\int }}\phi_{1,1}^{\prime \prime }\phi_{1,1}dx\overset{1}{\underset{0}{\int }}\phi_{1,1}^{\prime }w_{s,1}^{\prime }dx-12\alpha_{2}V_{\mathrm{dc},1}^{2}\overset{1}{\underset{0}{\int }}H_{1}\phi_{1,1}^{3}w_{s,1}dx\\ \kappa_{13}= & -4\alpha_{2}V_{\mathrm{dc},1}^{2}\overset{1}{\underset{0}{\int }}H_{1}\phi_{1,1}^{4}dx+\alpha_{1}\overset{1}{\underset{0}{\int }}\phi_{1,1}^{\prime \prime }\phi_{1,1}dx\overset{1}{\underset{0}{\int }}{\left(\phi_{1,1}^{\prime }\right)}^{2}dx\\ \kappa_{c1}= & \int_{0}^{1}-V_{c}^{2}\alpha_{2}H_{2}\phi_{1,1}\phi_{1,1}dx\\ \kappa_{c2}= & \int_{0}^{1}V_{c}^{2}\alpha_{2}H_{2}\phi_{1,1}\phi_{2,1}dx\\ f_{1}= & 2\alpha_{2}V_{\mathrm{dc},1}V_{\mathrm{ac},1}\int_{0}^{1}H\left(1+2w_{s,1}+3{w_{s,1}}^{2}+4{w_{s,1}}^{3}\right)\phi_{1,1}dx\\ \kappa_{21}= & \int_{0}^{1}\frac{{h_{2}}^{2}\phi_{2,1}{\phi_{2,1}}^{\left(4\right)}}{{h_{1}}^{2}}dx \end{array}$$
